# Association between alopecia areata and cardiovascular disease: a systematic review and meta-analysis

**DOI:** 10.3389/fimmu.2025.1643709

**Published:** 2025-08-07

**Authors:** Jiawei Lu, Xuechen Cao, Yifei Feng, Yongkai Yu, Yan Lu

**Affiliations:** Department of Dermatology, The First Affiliated Hospital with Nanjing Medical University, Nanjing, China

**Keywords:** alopecia areata, cardiovascular disease risk, cytokine, systematic review, inflammation

## Abstract

**Background:**

Alopecia areata (AA) is a common autoimmune disorder causing patchy hair loss. Epidemiological observations and molecular studies collectively suggest an underrecognized interplay between AA and cardiovascular disease (CVD). However, the relationship between them remains controversial and requires further investigation.

**Objective:**

To evaluate the association between AA and CVD through a meta-analysis of combinable results.

**Methods:**

We systematically searched four databases (MEDLINE, Embase, Web of Science, and Cochrane Library) for relevant studies from inception to December 6, 2024. Studies included in the analysis were cohort or case-control studies that focused on the relationship between AA and CVD. Two independent reviewers extracted the data. The study quality was evaluated using the Newcastle-Ottawa Scale. A random-effects model was used for meta-analysis to calculate the odds ratio (OR) and 95% confidence intervals (CIs).

**Results:**

Our search yielded five studies involving 238,270 AA patients from three countries. The meta-analysis revealed that AA patients had an increased OR (OR = 1.71; 95% CI: 1.0 to 2.92; *p* < 0.01) for CVD outcomes compared to the control group. Subgroup analysis revealed a stronger risk in patients with alopecia totalis or alopecia universalis (OR = 3.80; 95% CI: 1.65 to 8.73; *p* < 0.01). Associations were not observed between patch-type AA and CVD, nor between AA and ischemic stroke or myocardial infarction.

**Conclusions:**

This meta-analysis suggests that AA patients, especially those with alopecia totalis or alopecia universalis, may have an elevated risk of developing CVD. Given the shared immunological mechanisms, systemic inflammation in AA may contribute to the development of atherosclerosis and increased cardiovascular risk. Further studies are needed to validate these findings and clarify the underlying mechanisms.

## Introduction

Alopecia areata (AA) is an autoimmune disorder characterized by non-scarring alopecia. AA prevalence varies between 0.02% and 0.21% (1), with adults being more commonly affected than children, and women having a higher incidence than men (2, 3). Several studies have revealed a modest increase over time in the incidence of AA (4–7). Alopecia totalis (AT) and alopecia universalis (AU), characterized by the complete loss of scalp hair and body hair, respectively, represent the severe subtypes of AA and are associated with more pronounced clinical features and a poorer prognosis. The course of AA is often unpredictable and persistent, especially in patients with extensive hair loss. This can lead to stigma, psychosocial distress, reduced work productivity, and lower quality of life (8).

Recently, several cohort studies have verified comorbidities in patients with AA, including skin diseases, inflammatory diseases, cancer, gastrointestinal diseases, viral infection, ocular and auditory disorders, thyroid diseases, and mental disorders. A potential correlation between AA and cardiovascular disease (CVD) has also been reported. The levels of cardiac troponin I and CRP in the plasma of patients with AA were found to be higher than those of individuals with androgenetic alopecia and no hair loss (9, 10). A proteomic analysis of AA blood profiles revealed a higher inflammatory microenvironment in the peripheral blood of AA patients, as well as significant enrichment in atherosclerosis signaling pathways. Correlation analysis showed that the cardiovascular/atherosclerosis and immune pathways were significantly correlated with Severity of Alopecia Tool (SALT) in patients with AA (r > 0.35; *p* < 0.05) (11). The serum concentration of chemokine C-C motif ligand 7, a known biomarker for atherosclerosis, was found to be positively correlated with the severity of AA (12).

However, the potential mechanisms underlying the link between AA and CVD remain unknown. In AA, the collapse of hair follicle immune privilege is considered a critical initiating event, particularly in genetically predisposed individuals. This process is driven by CD8^+^ NKG2D^+^ T cells around hair follicles alongside a spectrum of inflammatory cells. CD8^+^ NKG2D^+^ T cells produce IFN-γ via JAK1/2 signaling, which induces IL-15 release from follicular cells. IL-15 then further enhances IFN-γ production through JAK1/3 on CD8^+^ NKG2D^+^ T cells, forming a positive feedback loop. The coordinated interaction of immune cells around hair bulbs highlights the complex immunological landscape of AA, involving not only perifollicular CD4^+^ T cells (13) but also regulatory T cells, dendritic cells, dendritic epidermal T cells, natural killer T cells, tissue‐resident memory T cells, mast cells, and eosinophils. In the cardiovascular system, CD8^+^ T cell infiltration has long been recognized in both human and murine atherosclerotic vessels (14). Clinical and experimental evidence indicates that CD8^+^ T cells contribute to endothelial injury and atherosclerotic lesion progression through multiple mechanisms, including the induction of endothelial cell apoptosis, the regulation of monocyte recruitment via IFN-γ–dependent bone marrow monopoiesis, and the promotion of necrotic core growth through the production of perforin, Granzyme B, and TNF-α (14–16). In patients with acute ischemia, enrichment of both CD4^+^ and CD8^+^ T cells was found near intact fibrous caps (17), which promotes tissue damage and remodeling by releasing cytotoxic molecules such as Granzyme B, perforin, or granulysin (17, 18). The proatherogenic role of conventional type 1 dendritic cells has also been recently discovered in atherosclerosis by enhancing CD4^+^ and CD8^+^ T-cell immunity in low-density lipoprotein receptor-deficient (Ldlr^−^/^−^) mice fed a high-cholesterol diet (19), serving as a bridge between innate and adaptive immunity.

Controversial results of population-based cohort studies have been published regarding the relationship between CVD and AA patients (19–22). Whether patients with AA are at an elevated risk of CVD compared to the healthy population remains unclear, highlighting the need for a thorough synthesis of existing studies. Therefore, the present study aims to systematically review and summarize current evidence on the association between AA and CVD, to clarify their potential link and provide implications for clinical decision-making and healthcare planning.

## Materials and methods

This meta-analysis was performed in accordance with the Preferred Reporting Items for Systematic Reviews and Meta-Analysis (PRISMA) (23) reporting guideline (**Supplementary Table S1**). The protocol for this study was registered with PROSPERO (CRD42024627718) at https://www.crd.york.ac.uk/PROSPERO.

### Data source and search strategy

A time-limited search was conducted from inception to December 6, 2024, on four databases: MEDLINE, Embase, Web of Science, and the Cochrane Library. The search strategy in each database is detailed in **Supplementary Table S2**. After removing duplicate documents, two reviewers (J.W. Lu and X.C. Cao) independently screened titles and abstracts for eligibility. Additionally, we manually searched the reference lists of included studies to identify any further relevant research. Subsequently, the full texts were read for the final selection. Studies were included if they described the association between AA and CVD through randomized controlled trials or observational studies, involved human participants, and reported measurable CVD outcomes. Only studies that provided quantifiable data (such as odds ratios, relative risks, or hazard ratios) for inclusion in the meta-analysis were considered. Excluded studies included reviews, conference papers, case reports, animal studies, and those without quantifiable data on AA and CVD. The final selection of studies was determined after discussion between the two reviewers, with a third reviewer involved to resolve any disputes. Five studies were ultimately included for further analysis.

### Data extraction and quality assessment

Two reviewers (J.W. Lu and X.C. Cao) independently verified the data extracted from each study, including the study design, sample size, participant demographics (age, gender, and race/ethnicity), CVD outcomes (e.g., myocardial infarction, ischemic stroke) and other risk factors (e.g., smoking status, comorbidities). The studies were evaluated for quality using the Newcastle-Ottawa Scale (24) (**Table 1**), with scores of 7 or higher indicating a high quality of the work. Newcastle–Ottawa Scale (NOS).

**Table 1:** Quality of evidence in included studies in the systematic review.

Study	Selection				Comparability	Outcome			Score
Longitudinal Studies	Representativeness of the exposed cohort	Selection of the non-exposed cohort	Ascertainment of exposure	Demonstration that outcome of interest was not present at start of study	Comparability of cohorts based on the design or analysis	Assessment of outcome	Was follow-up long enough for outcomes to occur?	Adequacy of follow up of cohorts	0 to 9
Kang et al.	1	1	1	1	2	1	1	1	9
Huang et al.	1	1	1	1	2	1	0	1	8
Shin et al.	1	1	1	1	2	1	1	1	9
Lee et al.	1	1	1	1	2	1	1	1	9
Caldas et al.	1	1	1	1	1.5	1	0	1	7.5

A score system was used to perform a semiquantitative assessment of the study quality. NOS ranges from 0 to 9 scores. We deemed high-quality studies that achieved 7scores, medium-quality studies 4 to 6 scores, and poor-quality studies < 4 scores. NOS, Newcastle–Ottawa Scale.

### Data synthesis and statistical analysis

Analysis was performed using R software (version 4.3.2). Study-specific risk estimates were pooled by using random effects meta-analyses because I² > 50%. Subgroup analyses were stratified by CVD subtypes (ischemic stroke and myocardial infarction) and AA subtypes (patch-type AA and AT/AU). The meta-analysis protocol, including literature search strategies, data extraction, and statistical methods, was strictly aligned with the MOOSE checklist for observational studies (25).

## Results

### Study selection

**Figure 1:**
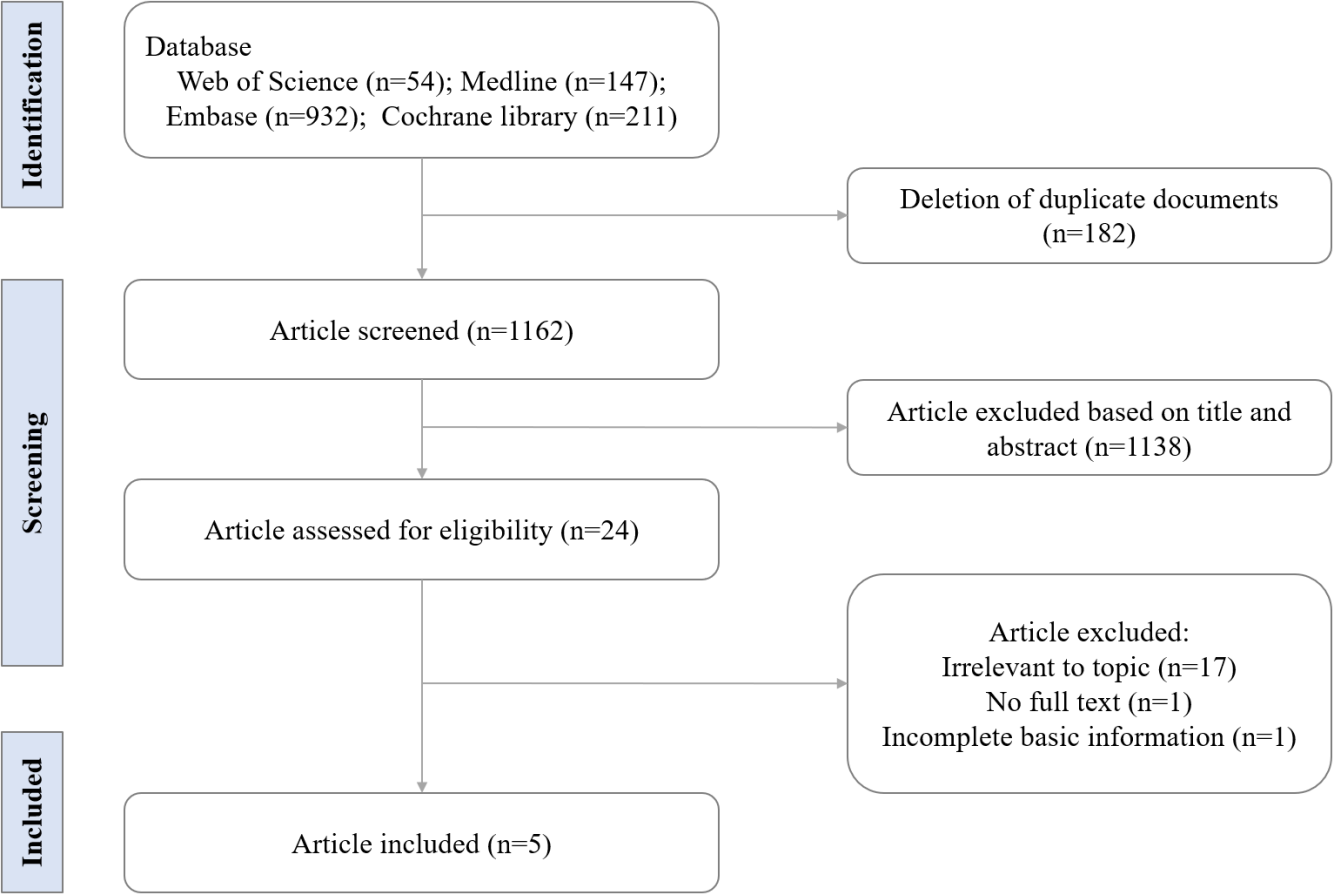
Literature search and study selection process.

Our initial database search identified 1344 records. After excluding 1320 records, which included duplicated documents, animal studies, and non-research articles (e.g., case reports, conference documents, and reviews), 24 full-text articles were assessed for eligibility. In total, five studies involving 238,270 AA patients from three different countries were included (**Figure 1**). Of these, four were retrospective cohort studies and one was a matched case-control study. The sample sizes in the AA group ranged from 1006 to 228,886. Further details regarding the characteristics of these studies are presented in **Table 2**.

**Table 2:** Characteristics of included studies.

Authors (Publication Year, Country/Region)	Study Design	Study Period	Number of Controls (M/F/Others)	Numbers of Cases (M/F/Others)	Mean Age of Controls, Years, Mean (SD)	Mean Age of Cases, Years, Mean (SD)	Classification of AA (Number of Cases)	Outcome
Kang et al. (2015, Taiwan)	Retrospective Cohort Study	3 Years	16155(7940/8215)	3231(1588/1643)	NA	NA	NA	Hemorrhagic Stroke, Ischemic Stroke, Unspecified Stroke
Huang et al. (2016, US)	Propensity-Matched Retrospective Analysis	NA	4131(1625/2506)	1377(517/860)	44.4(15.3)	45.0(15.7)	NA	Ischemic Stroke, Acute Myocardial Infarction
Shin et al. (2020, Korea)	Retrospective Cohort Study	January 2002 to December 2017	4577720(2551280/2026440)	228886(127564/101322)	NA	NA	AT (12131) AU (7765) Patch-Type AA (208990)	Acute Myocardial Infarction
Lee et al. (2021, Korea)	Retrospective Cohort Study	12 Years	18850(8310/10540)	3770(1662/2108)	NA	NA	Mild (3664) Severe (106)	Heart Failure, Angina Pectoris, Acute Myocardial Infarction, Chronic Myocardial Infarction
Caldas et al. (2024, US)	Case-Control Study	NA	4024(888/3028/108)	1006(229/751/26)	55.84(15.23)	55.94(15.67)	AA (930) AT or AU (76)	Ischemic Heart Disease, Cerebral Vascular Disease

SD, standard deviation; AA alopecia areata; AT, alopecia totalis; AU, alopecia universalis; NA, not applicable.

### Primary outcomes

**Figure 2:**
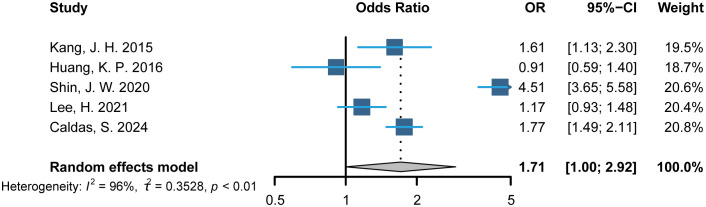
Primary analysis: Composite of alopecia areata and cardiovascular disease. OR indicates odds ratio.

This meta-analysis included data from five relevant studies (19–22, 26). After detecting heterogeneity, we used a random-effects model and combined the results of the trials. The meta-analysis indicated that the AA group had an increased odds ratio (OR) for CVD, with a value of 1.71(95% CI: 1.0 to 2.92; *p*<0.01), compared to individuals without AA (**Figure 2**).

### Subgroup analysis

#### Subtypes of AA

**Figure 3:**
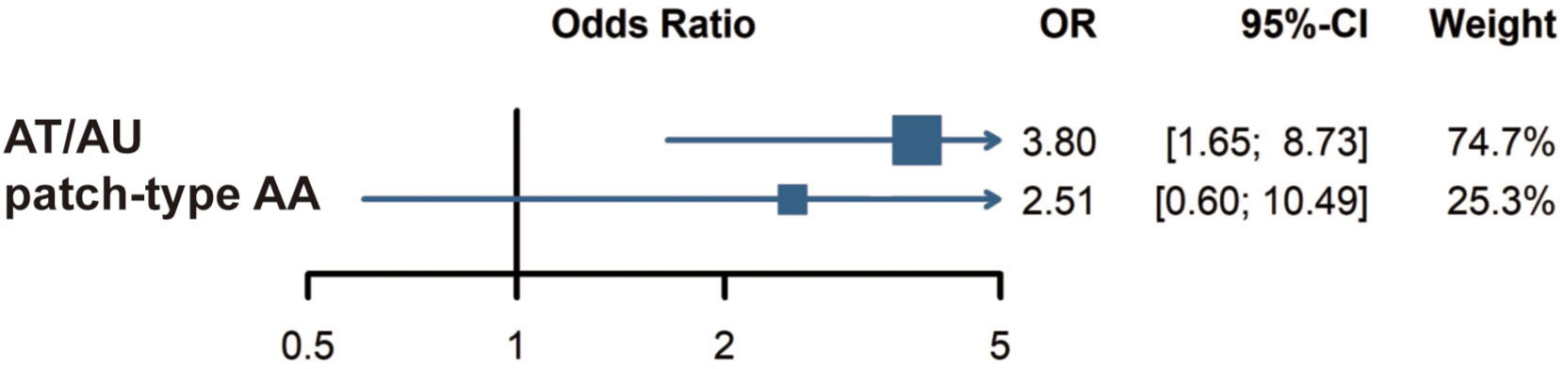
Subgroup analysis: Risk of cardiovascular disease in AT/AU and patch-type AA patients. AT, alopecia totalis; AU, alopecia universalis; AA, alopecia areata.

Three of the five studies involved data on AA subtypes. One study defined severe AA as AT, AU, or ophiasis. However, no CVD events were observed in the group of severe AA in that study; therefore, it was excluded from our subgroup analysis. Two other studies categorized AA patients into two groups: those with patch-type AA and those with AT/AU, and were thus included in the subgroup analysis. A meta-analysis of these studies revealed a stronger association between AT/AU and CVD (OR = 3.80; 95% CI: 1.65 to 8.73; *p* = 0.0017) compared to the healthy population (**Figure 3**). Association between patch-type AA and CVD was not observed (*p* = 0.6241).

#### Subtypes of CVD

**Figure 4:**
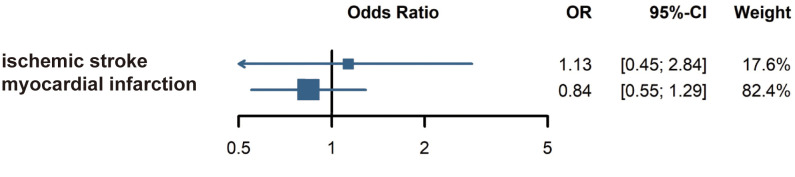
Subgroup analysis: Risk of ischemic stroke and myocardial infarction in alopecia areata patients.

The five articles employed different classification methods for CVD, including distinctions between hemorrhagic and ischemic, acute and chronic, and cerebral and cardiac types. This inconsistency in CVD classification may have influenced the results. Subgroup analysis of ischemic stroke and myocardial infarction found no significant associations between AA and those conditions, as shown in **Figure 4**.

## Discussion

AA is a chronic autoimmune disease that destroys the hair follicles, leading to patchy hair loss on the scalp and other hair-bearing areas. The course of AA is often unpredictable, and patients experience great stress, anxiety, and impaired health-related quality of life throughout the disease course (27). Recently, retrospective cohort studies on the association between AA and CVD have been published, but their conclusions are controversial. To the best of our knowledge, this is the first meta-analysis to systematically examine this relationship.

There have been a few reports on the potential link in clinical and animal studies. The plasma level of cardiac troponin I, a biomarker of myocardial ischemia and inflammation, was higher in subjects with AA than in those without hair loss, with the highest levels found in untreated subjects (9). In addition, the serum level of N-terminal pro-B-type natriuretic peptide, used in the diagnosis of congestive heart failure, was found to be higher in AA patients than in healthy populations (10). In a cross-sectional study, four cardiovascular-risk proteins (OLR1/OSM/MPO/PRTN3) and five cardiovascular/atherosclerosis-related proteins (SELP/PGLYRP1/MPO/IL-18/OSM) were significantly differentially expressed between AA and control individuals (11). AA severity was associated with a higher risk of arterial stiffness (28). In a C3H/HeJ mouse model of AA, atrial and ventricular hypertrophy, as well as collagen deposition, were observed in comparison to the control group (29).

Our findings indicated that patients with AA are at an elevated risk of developing CVD compared to individuals without AA. AT and AU are severe subtypes of AA and are typically more refractory to treatment and remission (30). Subgroup analysis revealed a stronger association between AT/AU and CVD. This finding is consistent with the results of a cross-sectional study, which showed that patients with AT/AU exhibited a higher inflammatory state, increased cardiovascular risk, and atherosclerosis-linked biomarkers in their blood compared to control individuals and patients with two other immune-mediated skin diseases (11). Given the higher immune alterations in AA scalp (31) and the correlation between its clinical severity and biomarkers of immune and cardiovascular dysregulation (32), early systemic treatments are highly recommended in patients with significant AA involvement.

AA and CVD potentially share some common immunological mechanisms, where immune dysregulation in follicular hair epithelium beyond the scalp may contribute to the circulatory abnormalities. In both conditions, CD8^+^ T cells are crucial, with their activation driving inflammation. In AA, CD8^+^ NKG2D^+^ T cells mediate the disruption of hair follicle immune privilege, leading to subsequent immune cell recruitment, while in CVD, CD8^+^ T cell infiltration contributes to endothelial injury and atherosclerosis progression. Both conditions also involve the release of pro-inflammatory cytokines like IFN-γ and TNF-α, and the activation of various innate and adaptive immune cells, such as CD4^+^ T cells, T helper (Th) 1, Th2, Th17 cells, and dendritic cells, which initiate and amplify the inflammatory response and tissue damage (33–37). Recently, a higher value of pan CD4^+^T cells was found to be associated with lower endothelial function in participants of the Multi-Ethnic Study of Atherosclerosis (34). Th1 cells play a pro-atherosclerotic role through the secretion of pro-inflammatory cytokines, including IFN-γ, TNF-α, IL-2, IL-3, and lymphotoxin, which sustain chronic inflammation, accelerate foam cell formation, and impair endothelial integrity (38). In a murine model of atherosclerosis, neutrophil extracellular traps activated macrophages, which in turn produced cytokines that activated Th17 cells. These cells add to immune cell recruitment in atherosclerotic plaques (39). Cells positive for IL-17 were detected in the aortic sinus of *Ldlr* ^−/−^ mice (40) and the aortas of apolipoprotein E-deficient (*Apoe* ^−/−^) mice (41). Furthermore, elevated populations of Th17 and γδIL-17^+^ T cells were observed in the aortic wall, aortic adventitia, and secondary lymphoid organs of aged *Apoe* ^−/−^ and C57/BL6 mice (42).

However, no study has directly identified that immune dysregulation in AA patients plays a pathogenic role in CVD. Furthermore, research indicates that the pathogenesis of different CVD subtypes varies, and AA patients may exhibit varying risks for different types of CVD. In our study, we extracted subcategories across the studies and performed meta-analyses for ischemic stroke and myocardial infarction in AA patients. No significant association was identified between AA and ischemic stroke or myocardial infarction. Similar findings were reported in a meta-analysis that included only one relevant study (43). Considering the limited number of published studies on this topic, this conclusion still requires further research in the future.

In the included studies, only Jung-Won Shin et al. conducted a subgroup analysis based on age, gender and smoking status. The results showed that men with AA had an earlier and significantly higher incidence of acute myocardial infarction compared to women, with a similar pattern in individuals under 50 years of age. Although the prevalence of AA is higher in women, male AA patients may have a higher risk of CVD. Furthermore, smoking enhanced the association between AA and myocardial infarction, with smokers experiencing a higher risk of myocardial infarction at an earlier time point than non-smokers. It has been reported that smoking can exacerbate inflammation by affecting immune effector cells, such as macrophages, neutrophils, and T lymphocytes, which may be one of the potential reasons for the higher CVD risk in smoking AA patients (44). Heera Lee’s study conducted a subgroup analysis based on disease duration, which showed that the hazard ratios for CVD in both mild and episodic AA patients were higher than those in matched controls (mild AA, *p* = 0.110; episodic AA, *p* = 0.113). In contrast, the hazard ratio for CVD events in long-standing AA patients was lower than in controls (*p* = 0.738). These findings warrant further investigation.

## Limitations

Our study has certain limitations. Firstly, the included studies were limited to those from the USA, Taiwan, and Korea, with no data from research conducted in African and European countries. This lack of global representation may limit the generalizability of the findings to other populations. Secondly, the quality of the included studies varied, and several had relatively small sample sizes, which may affect the robustness and reliability of the pooled estimates. Thirdly, the diagnosis of patch-type AA, AT, and AU relied on the clinical judgment of dermatologists, which is inherently subjective and may affect the consistency of the results. Additionally, the subtypes of CVD included in the studies were not fully consistent, and different subtypes may have distinct clinical characteristics and risks. Finally, due to the limited published data, a more comprehensive analysis of potential differences related to age, gender, and other relevant factors could not be performed. Considering these factors contributing to heterogeneity, there is an urgent need for large-scale, high-quality, prospective cohort studies with standardized diagnostic criteria and sufficient adjustment for confounding variables to validate the observed associations and clarify causality.

## Conclusions

In summary, individuals with AA face an increased risk of developing CVD, with a more pronounced association observed in cases of AT or AU. Healthcare providers should be aware of this potential risk and take proactive measures to mitigate comorbidities. A cardiovascular risk assessment is highly recommended in patients with severe clinical presentations of AA. Further studies are needed with larger sample sizes and more diverse populations. These studies should include standardized subtype classifications for AA and CVD, as well as perform subgroup analyses on relevant covariates, such as age, gender, smoking status, and comorbidities, to reduce heterogeneity and improve the precision of findings.

## References

[1] Jeon JJ Jung SW Kim YH Parisi R Lee JY Kim MH. Global, regional and national epidemiology of alopecia areata: a systematic review and modelling study. Br J Dermatol. (2024) 191:325–35. doi: 10.1093/bjd/ljae058, PMID: 38332643

[2] Jacobsen EW Pedersen OB Andorsdottir G Jemec GBE Bryld LE. Family recurrence risk of alopecia areata in the Faroe Islands. Clin Exp Dermatol. (2019) 44:e224–e9. doi: 10.1111/ced.13974, PMID: 30929273

[3] Harries M Macbeth AE Holmes S Chiu WS Gallardo WR Nijher M. The epidemiology of alopecia areata: a population-based cohort study in UK primary care. Br J Dermatol. (2022) 186:257–65. doi: 10.1111/bjd.20628, PMID: 34227101 PMC9298423

[4] Lee JH Kim HJ Han KD Han JH Bang CH Park YM. Incidence and prevalence of alopecia areata according to subtype: a nationwide, population-based study in South Korea (2006-2015). Br J Dermatol. (2019) 181:1092–3. doi: 10.1111/bjd.18145, PMID: 31102412

[5] Soh BW Kim SM Kim YC Choi GS Choi JW. Increasing prevalence of alopecia areata in South Korea. J Dermatol. (2019) 46:e331–e2. doi: 10.1111/1346-8138.14863, PMID: 30908740

[6] Mostaghimi A Gao W Ray M Bartolome L Wang T Carley C. Trends in prevalence and incidence of alopecia areata, alopecia totalis, and alopecia universalis among adults and children in a US employer-sponsored insured population. JAMA Dermatol. (2023) 159:411–8. doi: 10.1001/jamadermatol.2023.0002, PMID: 36857069 PMC9979012

[7] Ali NS Tollefson MM Lohse CM Torgerson RR. Incidence and comorbidities of pediatric alopecia areata: A retrospective matched cohort study using the Rochester Epidemiology Project. J Am Acad Dermatol. (2022) 87:427–9. doi: 10.1016/j.jaad.2021.08.050, PMID: 34487778 PMC9815486

[8] Muntyanu A Gabrielli S Donovan J Gooderham M Guenther L Hanna S. The burden of alopecia areata: A scoping review focusing on quality of life, mental health and work productivity. J Eur Acad Dermatol Venereol. (2023) 37:1490–520. doi: 10.1111/jdv.18926, PMID: 36708097

[9] Wang EH Santos L Li XY Tran A Kim SSY Woo K. Alopecia areata is associated with increased expression of heart disease biomarker cardiac troponin I. Acta Dermato-Venereol. (2018) 98:776–82. doi: 10.2340/00015555-2964, PMID: 29740659

[10] El-Sayed Mahmoud Marie R El-Sayed GAK Attia FM Gomaa AHA. Evaluation of serum cardiac troponin I and N-terminal pro-B-type natriuretic peptide levels in patients with alopecia areata. Clin Exp Dermatol. (2021) 46:153–6. doi: 10.1111/ced.14425, PMID: 32810890

[11] Glickman JW Dubin C Renert-Yuval Y Dahabreh D Kimmel GW Auyeung K. Cross-sectional study of blood biomarkers of patients with moderate to severe alopecia areata reveals systemic immune and cardiovascular biomarker dysregulation. J Am Acad Dermatol. (2021) 84:370–80. doi: 10.1016/j.jaad.2020.04.138, PMID: 32376430

[12] Waskiel-Burnat A Niemczyk A Blicharz L Chmielinska P Zaremba M Gasecka A. Chemokine C-C motif ligand 7 (CCL7), a biomarker of atherosclerosis, is associated with the severity of alopecia areata: A preliminary study. J Clin Med. (2021) 10(22):5418. doi: 10.3390/jcm10225418, PMID: 34830700 PMC8624305

[13] Todes-Taylor N Turner R Wood GS Stratte PT Morhenn VB. T cell subpopulations in alopecia areata. J Am Acad Dermatol. (1984) 11:216–23. doi: 10.1016/s0190-9622(84)70152-6, PMID: 6384283

[14] Cochain C Koch M Chaudhari SM Busch M Pelisek J Boon L. CD8+ T cells regulate monopoiesis and circulating ly6C-high monocyte levels in atherosclerosis in mice. Circ Res. (2015) 117:244–53. doi: 10.1161/CIRCRESAHA.117.304611, PMID: 25991812

[15] Kyaw T Winship A Tay C Kanellakis P Hosseini H Cao A. Cytotoxic and proinflammatory CD8+ T lymphocytes promote development of vulnerable atherosclerotic plaques in apoE-deficient mice. Circulation. (2013) 127:1028–39. doi: 10.1161/CIRCULATIONAHA.112.001347, PMID: 23395974

[16] Winkels H Ehinger E Vassallo M Buscher K Dinh HQ Kobiyama K. Atlas of the immune cell repertoire in mouse atherosclerosis defined by single-cell RNA-sequencing and mass cytometry. Circ Res. (2018) 122:1675–88. doi: 10.1161/CIRCRESAHA.117.312513, PMID: 29545366 PMC5993603

[17] Leistner DM Krankel N Meteva D Abdelwahed YS Seppelt C Stahli BE. Differential immunological signature at the culprit site distinguishes acute coronary syndrome with intact from acute coronary syndrome with ruptured fibrous cap: results from the prospective translational OPTICO-ACS study. Eur Heart J. (2020) 41:3549–60. doi: 10.1093/eurheartj/ehaa703, PMID: 33080003 PMC7780480

[18] Santos-Zas I Lemarie J Zlatanova I Cachanado M Seghezzi JC Benamer H. Cytotoxic CD8(+) T cells promote granzyme B-dependent adverse post-ischemic cardiac remodeling. Nat Commun. (2021) 12:1483. doi: 10.1038/s41467-021-21737-9, PMID: 33674611 PMC7935973

[19] Shin JW Kang T Lee JS Kang MJ Huh CH Kim MS. Time-dependent risk of acute myocardial infarction in patients with alopecia areata in korea. JAMA Dermatol. (2020) 156:763–71. doi: 10.1001/jamadermatol.2020.1133, PMID: 32401269 PMC7221866

[20] Lee H Kim YC Choi JW. Alopecia areata is not a risk factor for heart diseases: A 10-year retrospective cohort study. PLoS One. (2021) 16:e0250216. doi: 10.1371/journal.pone.0250216, PMID: 33961663 PMC8104430

[21] Kang JH Lin HC Kao S Tsai MC Chung SD. Alopecia areata increases the risk of stroke: a 3-year follow-up study. Sci Rep. (2015) 5:11718. doi: 10.1038/srep11718, PMID: 26114569 PMC4481771

[22] Huang KP Joyce CJ Topaz M Guo Y Mostaghimi A. Cardiovascular risk in patients with alopecia areata (AA): A propensity-matched retrospective analysis. J Am Acad Dermatol. (2016) 75:151–4. doi: 10.1016/j.jaad.2016.02.1234, PMID: 27183846

[23] Liberati A Altman DG Tetzlaff J Mulrow C Gotzsche PC Ioannidis JP. The PRISMA statement for reporting systematic reviews and meta-analyses of studies that evaluate healthcare interventions: explanation and elaboration. BMJ. (2009) 339:b2700. doi: 10.1136/bmj.b2700, PMID: 19622552 PMC2714672

[24] Stang A. Critical evaluation of the Newcastle-Ottawa scale for the assessment of the quality of nonrandomized studies in meta-analyses. Eur J Epidemiol. (2010) 25:603–5. doi: 10.1007/s10654-010-9491-z, PMID: 20652370

[25] Stroup DF Berlin JA Morton SC Olkin I Williamson GD Rennie D. Meta-analysis of observational studies in epidemiology: a proposal for reporting. Meta-analysis Of Observational Studies in Epidemiology (MOOSE) group. JAMA. (2000) 283:2008–12. doi: 10.1001/jama.283.15.2008, PMID: 10789670

[26] Caldas S Pagan AD Pulsinelli J da Rosa JC Jung S Sharma D. Association of alopecia areata and ischemic heart disease and cerebrovascular in U.S. adults: an all of us database study. Arch Dermatol Res. (2024) 316:202. doi: 10.1007/s00403-024-02959-5, PMID: 38775975

[27] Tan IJ Jafferany M. Psychosocial impact of alopecia areata in paediatric and adolescent populations: A systematic review. J Paediatr Child Health. (2024) 60:778–82. doi: 10.1111/jpc.16678, PMID: 39376033 PMC11616254

[28] Najafi MT Abedini R Ghandi N Seraji S Sadeghi Y. Is the severity of alopecia areata associated with arterial stiffness? J Res Med Sci. (2023) 28:80. doi: 10.4103/jrms.jrms_375_23, PMID: 38292334 PMC10826848

[29] Wang E Chong K Yu M Akhoundsadegh N Granville DJ Shapiro J. Development of autoimmune hair loss disease alopecia areata is associated with cardiac dysfunction in C3H/HeJ mice. PLoS One. (2013) 8:e62935. doi: 10.1371/journal.pone.0062935, PMID: 23658656 PMC3637254

[30] Choi SM Kang MJ Kwon SH Sim WY Lew BL. A retrospective study on the clinical characteristics and prognosis of alopecia totalis and universalis: An update on prognosis. J Dermatol. (2023) 50:1335–8. doi: 10.1111/1346-8138.16840, PMID: 37208851

[31] Suarez-Farinas M Ungar B Noda S Shroff A Mansouri Y Fuentes-Duculan J. Alopecia areata profiling shows TH1, TH2, and IL-23 cytokine activation without parallel TH17/TH22 skewing. J Allergy Clin Immunol. (2015) 136:1277–87. doi: 10.1016/j.jaci.2015.06.032, PMID: 26316095

[32] Song T Pavel AB Wen HC Malik K Estrada Y Gonzalez J. An integrated model of alopecia areata biomarkers highlights both T(H)1 and T(H)2 upregulation. J Allergy Clin Immunol. (2018) 142:1631–4 e13. doi: 10.1016/j.jaci.2018.06.029, PMID: 29981808

[33] Van Acker MM Schwartz RR Andrews K Seiffert-Sinha K Sinha AA. Inheritance-specific dysregulation of th1- and th17-associated cytokines in alopecia areata. Biomolecules. (2023) 13(9):1285. doi: 10.3390/biom13091285, PMID: 37759685 PMC10527519

[34] DeConne TM Sitlani CM Decker KP Delaney JA Psaty BM Doyle MF. Associations of circulating T cell subsets with endothelial function: the Multi-Ethnic Study of Atherosclerosis. Am J Physiol Heart Circ Physiol. (2025) 328:H1374–H9. doi: 10.1152/ajpheart.00893.2024, PMID: 39930879 PMC12166541

[35] Allam G Abdel-Moneim A Gaber AM. The pleiotropic role of interleukin-17 in atherosclerosis. BioMed Pharmacother. (2018) 106:1412–8. doi: 10.1016/j.biopha.2018.07.110, PMID: 30119214

[36] Li Q Liu M Fu R Cao Q Wang Y Han S. Alteration of circulating innate lymphoid cells in patients with atherosclerotic cerebral infarction. Am J Transl Res. (2018) 10:4322–30., PMID: 30662674 PMC6325527

[37] Inui H Nishida M Ichii M Nakaoka H Asaji M Ide S. XCR1(+) conventional dendritic cell-induced CD4(+) T helper 1 cell activation exacerbates cardiac remodeling after ischemic myocardial injury. J Mol Cell Cardiol. (2023) 176:68–83. doi: 10.1016/j.yjmcc.2023.01.011, PMID: 36739942

[38] Koltsova EK Garcia Z Chodaczek G Landau M McArdle S Scott SR. Dynamic T cell-APC interactions sustain chronic inflammation in atherosclerosis. J Clin Invest. (2012) 122:3114–26. doi: 10.1172/JCI61758, PMID: 22886300 PMC3428082

[39] Warnatsch A Ioannou M Wang Q Papayannopoulos V. Inflammation. Neutrophil extracellular traps license macrophages for cytokine production in atherosclerosis. Science. (2015) 349:316–20. doi: 10.1126/science.aaa8064, PMID: 26185250 PMC4854322

[40] Romain M Ramkhelawon B Uyttenhove C Pasterkamp G Herbin O. Loss of SOCS3 expression in T cells reveals a regulatory role for interleukin-17 in atherosclerosis. J Exp Med. (2009) 206:2067–77. doi: 10.1084/jem.20090545, PMID: 19737863 PMC2757872

[41] Ma T Gao Q Zhu F Guo C Wang Q Gao F. Th17 cells and IL-17 are involved in the disruption of vulnerable plaques triggered by short-term combination stimulation in apolipoprotein E-knockout mice. Cell Mol Immunol. (2013) 10:338–48. doi: 10.1038/cmi.2013.4, PMID: 23542316 PMC4003212

[42] Smith E Prasad KM Butcher M Dobrian A Kolls JK Ley K. Blockade of interleukin-17A results in reduced atherosclerosis in apolipoprotein E-deficient mice. Circulation. (2010) 121:1746–55. doi: 10.1161/CIRCULATIONAHA.109.924886, PMID: 20368519 PMC2929562

[43] Amamoto M Yamada T Hara K. Updated meta-analysis of the relation between heart disease and androgenic alopecia or alopecia areata. Australas Med J. (2018) 11:25–33. doi: 10.21767/amj.2017.3270

[44] Liu Y Lu L Yang H Wu X Luo X Shen J. Dysregulation of immunity by cigarette smoking promotes inflammation and cancer: A review. Environ Pollut. (2023) 339:122730. doi: 10.1016/j.envpol.2023.122730, PMID: 37838314

