# Supplementary material for: Association between alopecia areata and cardiovascular disease: a systematic review and meta-analysis
Source: Front Immunol. 2025 Aug 7;16:1643709. doi: 10.3389/fimmu.2025.1643709 (PMC12367736; doi:10.3389/fimmu.2025.1643709)
Supplement: Supplementary file 2 [file SupplementaryFile2.docx]

Supplementary Table S2. Search strategy (inception-2024.12.6)

| PubMed | | |
| --- | --- | --- |
| #1 | (“alopecia areata”[tiab] OR “alopecia circumscripta”[tiab] OR “alopecia totalis”[tiab] OR “alopecia universalis”[tiab] OR “area celsi”[tiab] OR “pelade*”[tiab] OR “nonscarring hair loss” [tiab] OR “scarring hair loss” [tiab] OR "Alopecia Areata"[Mesh]) |  |
| #2 | (cardiotoxic*[tiab] OR "cardiac toxic*"[tiab] OR "cardiovascular disease*"[tiab] OR "heart disease*"[tiab] OR "ischemic heart disease*"[tiab] OR "ischaemic heart disease*"[tiab] OR "coronary occlusive disease*"[tiab] OR "myocardial ischemia*"[tiab] OR "myocardial infarction*"[tiab] OR "cardiovascular stroke*"[tiab] OR "heart attack*"[tiab] OR "heart failure"[tiab] OR "heart infarction*"[tiab] OR "cardiac failure"[tiab] OR "myocardial failure"[tiab] OR "angina pectoris"[tiab] OR "myocardial ischemia"[tiab] OR "cardiac valve disease*"[tiab] OR "heart valve disease*"[tiab] OR "valvular heart disease*"[tiab] OR "rheumatic heart disease*"[tiab] OR "bouillaud disease"[tiab] OR "rheumatic valve disease*"[tiab] OR "rheumatic valvular disease*"[tiab] OR "essential hypertension"[tiab] OR "essential arterial hypertension"[tiab] OR "idiopathic hypertension"[tiab] OR "hypertensive disease*"[tiab] OR "hypertensive heart disease*"[tiab] OR "hypertensive renal disease*"[tiab] OR "hypertensive nephropath*"[tiab] OR "renal hypertension"[tiab] OR "renovascular hypertension"[tiab] OR "hypertensive kidney disease*"[tiab] OR "hypertensive organ damage"[tiab] OR "secondary hypertension"[tiab] OR "pulmonary embolism*"[tiab] OR "lung embolism*"[tiab] OR "pulmonary thromboembolism*"[tiab] OR "pulmonary heart disease*"[tiab] OR "cor pulmonale"[tiab] OR pericarditis[tiab] OR pleuropericarditis[tiab] OR "pericardial inflammation"[tiab] OR endocarditis[tiab] OR endocarditides[tiab] OR "endocardial inflammation"[tiab] OR myocarditis[tiab] OR myocarditides[tiab] OR "myocardial inflammation"[tiab] OR cardiomyopath*[tiab] OR myocardiopath*[tiab] OR "myocardial disease*"[tiab] OR "cardiac conduction disorder*"[tiab] OR "cardiac conduction defect*"[tiab] OR "heart conduction disorder*"[tiab] OR "cardiac arrest"[tiab] OR "heart arrest"[tiab] OR "cardiac arrhythmia*"[tiab] OR "heart arrhythmia*"[tiab] OR arrhythmia*[tiab] OR "paroxysmal tachycardia*"[tiab] OR "paroxysmal reciprocal tachycardia*"[tiab] OR tachycardia*[tiab] OR "atrial fibrillation*"[tiab] OR "arterial disease*"[tiab] OR "artery disease*"[tiab] OR arteriopathy[tiab] OR "cerebrovascular disease*"[tiab] OR "cerebrovascular disorder*"[tiab] OR "cerebrovascular occlusion*"[tiab] OR stroke*[tiab] OR "cerebrovascular accident*"[tiab] OR "brain infarction*"[tiab] hemorrhage*"[tiab] OR "brain vascular accident*"[tiab] OR "subarachnoid OR "subarachnoid haemorrhage*"[tiab] OR "cerebrovascular haemorrhage*"[tiab] OR "cerebrovascular hemorrhage*"[tiab] OR "brain hemorrhage*"[tiab] OR "brain haemorrhage*"[tiab] OR "cerebral hemorrhage*"[tiab] OR "cerebral haemorrhage*"[tiab] OR "intracerebral hemorrhage*"[tiab] OR "intracerebral haemorrhage*"[tiab] OR "cerebral infarction*"[tiab] OR "cerebral infarct*"[tiab] OR "subcortical infarct*"[tiab] OR "cerebral arterial occlusion*"[tiab] OR "cerebral arterial thrombosis"[tiab] OR "occlusive cerebrovascular disease*"[tiab] OR "cerebral artery occlusion*"[tiab] OR "cerebral arterial stenos*"[tiab] OR "cerebral artery stenos*"[tiab] OR arterioscleros*[tiab] OR atheroscleros*[tiab] OR "aortic aneurysm*"[tiab] OR "carotid artery aneurysm*"[tiab] OR "carotid aneurysm*"[tiab] OR aneurysm*[tiab] OR embolism*[tiab] OR "arterial embolism"[tiab] OR "artery embolism"[tiab] OR "arterial disease*"[tiab] OR "artery disease*"[tiab] OR "diseases of the arteries"[tiab] OR "peripheral arterial disease*"[tiab] OR "capillary leak*"[tiab] OR "capillary disease*"[tiab] OR microangiopath*[tiab] OR "microvascular disease*"[tiab] OR "microcirculatory disease*"[tiab] OR "circulatory system disease*"[tiab] OR "circulatory disease"[tiab] OR "circulatory diseases"[tiab] OR "vein disease*"[tiab] OR "venous disease*"[tiab] OR "venous disorder*"[tiab] OR "diseases of the veins"[tiab] OR "varicose vein*"[tiab] OR varicosis[tiab] OR thrombophlebitis[tiab] OR "peripheral vascular disease*"[tiab] OR "peripheral angiopath*"[tiab] OR "peripheral arteriopathy*"[tiab] OR "peripheral vascular disorder*"[tiab] OR "vein embolism"[tiab] OR "venous embolism"[tiab] OR phlebitis[tiab] OR "portal vein thrombos*"[tiab] OR phlebothrombos*[tiab] OR "venous thrombos*"[tiab] OR "deep vein thrombos*"[tiab] OR haemorrhoid*[tiab] OR hemorrhoid*[tiab] OR "esophageal varices"[tiab] OR "esophageal varix"[tiab] OR "esophagus varices"[tiab] OR "esophagus varix"[tiab] OR hypotension[tiab] OR "low blood pressure"[tiab] OR "bundle branch block*"[tiab] OR "fascicular block*"[tiab] OR "atrioventricular block*"[tiab] OR "Cardiovascular Diseases"[Mesh] OR "Cardiomyopathies"[Mesh] OR "Myocarditis"[Mesh] OR "Endocarditis"[Mesh] OR "Pericarditis"[Mesh] OR "Myocardial Ischemia"[Mesh] OR "Myocardial Infarction"[Mesh] OR "Heart Failure"[Mesh] OR "Angina Pectoris"[Mesh] OR "Myocardial Ischemia"[Mesh] OR "Heart Valve Diseases"[Mesh] OR "Rheumatic Heart Disease"[Mesh] OR "Essential Hypertension"[Mesh] OR "Hypertension"[Mesh] OR "Hypertensive Nephropathy" [Supplementary Concept] OR "Hypertension, Renal"[Mesh] OR "Hypertension, Renovascular"[Mesh] OR "Pulmonary Embolism"[Mesh] OR "Pulmonary Heart Disease"[Mesh] OR "Cardiomyopathies"[Mesh] OR "Cardiac Conduction System Disease"[Mesh] OR "Heart Arrest"[Mesh] OR "Arrhythmias, Cardiac"[Mesh] OR "Tachycardia, Paroxysmal"[Mesh] OR "Tachycardia"[Mesh] OR "Atrial Fibrillation"[Mesh] OR "Cerebrovascular Disorders"[Mesh] OR "Stroke"[Mesh] OR "Subarachnoid Hemorrhage"[Mesh] OR "Cerebral Hemorrhage"[Mesh] OR "Cerebral Infarction"[Mesh] OR "Arteriosclerosis"[Mesh] OR "Atherosclerosis"[Mesh] OR "Aortic Aneurysm"[Mesh] OR "Carotid Artery Injuries"[Mesh] OR "Aneurysm"[Mesh] OR "Embolism"[Mesh] OR "Arterial Occlusive Diseases"[Mesh] OR "Peripheral Arterial Disease"[Mesh] OR "Capillary Leak Syndrome"[Mesh] OR "Varicose Veins"[Mesh] OR "Thrombophlebitis"[Mesh] OR "Peripheral Vascular Diseases"[Mesh] OR "Phlebitis"[Mesh] OR "Venous Thrombosis"[Mesh] OR "Hemorrhoids"[Mesh] OR "Esophageal and Gastric Varices"[Mesh] OR "Hypotension"[Mesh] OR "Cardiotoxicity"[Mesh] OR "Bundle Branch Block"[Mesh]) |  |
| #3 | ("epidemiological study"[tiab] OR "epidemiological studies"[tiab] OR cohort*[tiab] OR "concurrent study"[tiab] OR "concurrent studies"[tiab] OR "incidence study"[tiab] OR "incidence studies"[tiab] OR "cross sectional study"[tiab] OR "cross sectional studies"[tiab] OR "cross sectional survey*"[tiab] OR "prevalence study"[tiab] OR "prevalence studies"[tiab] OR "case control"[tiab] OR "case controls"[tiab] OR "followup study"[tiab] OR "followup studies"[tiab] OR "follow up study"[tiab] OR "follow up studies"[tiab] OR "followed up"[tiab] OR followedup[tiab] OR longitudinal[tiab] OR prospective[tiab] OR retrospective[tiab] OR registry[tiab] OR registries[tiab] OR “controlled before-after studies”[tiab] OR Registries[mesh] OR "Epidemiologic Studies"[Mesh] OR "Case-Control Studies"[Mesh] OR "Retrospective Studies"[Mesh] OR "Cohort Studies"[Mesh] OR "Follow-Up Studies"[Mesh] OR "Longitudinal Studies"[Mesh] OR "Prospective Studies"[Mesh] OR "Controlled Before-After Studies"[Mesh] OR "Cross-Sectional Studies"[Mesh]) |  |
| #4 | (letter[ptyp] OR editorial[ptyp] OR comment[ptyp] OR news[ptyp] OR "Congress"[Publication Type] OR "Consensus Development Conference"[Publication Type] OR editorial[tiab] OR commentary[tiab] OR “conference abstract*”[tiab] OR “conference proceeding*”[tiab] OR “systematic review*”[ti] OR “meta-analysis”[ptyp] OR “meta-analysis”[ti] OR “meta analyses”[ti] OR "Review"[Publication Type] OR "Systematic Review"[Publication Type] OR “retracted publication”[ptyp] OR “retraction of publication”[ptyp] OR “retraction of publication”[tiab] OR “retraction notice”[ti] OR “retracted publication”[tiab] OR "Published Erratum"[Publication Type] OR Corrigenda[tiab] OR corrigendum[tiab] OR errata[tiab] OR erratum[tiab] OR protocol[ti] OR protocols[ti]) |  |
| #5 | (Animals[MeSH Terms]) NOT (Humans[MeSH Terms]) |  |
| #6 | (mice[tiab] OR mouse[tiab] OR rat[tiab] OR rats[tiab] OR dog[tiab] OR dogs[tiab] OR pig[tiab] OR pigs[tiab] OR swine[tiab] OR porcine*[tiab] OR rodent*[tiab] OR animal*[tiab]) |  |
| #7 | #1 AND #2 |  |
| #8 | #7 AND #3 NOT ( #4 AND #5 AND #6) | 147 |

| Web of Science | | |
| --- | --- | --- |
| #1 | TS=("alopecia areata" OR "alopecia circumscripta" OR "alopecia totalis" OR "alopecia universalis" OR "area celsi" OR pelade* OR "nonscarring hair loss" OR "scarring hair loss") |  |
| #2 | TS=(cardiotoxic* OR "cardiac toxic*" OR "cardiovascular disease*" OR "heart disease*" OR "ischemic heart disease*" OR "ischaemic heart disease*" OR "coronary occlusive disease*" OR "myocardial ischemia*" OR "myocardial infarction*" OR "cardiovascular stroke*" OR "heart attack*" OR "heart failure" OR "cardiac conduction disorder*" OR "cardiac arrhythmia*" OR stroke* OR atheroscleros* OR arterioscleros* OR hypertension* OR embolism* OR aneurysm* OR myocarditis* OR pericarditis* OR endocarditis* OR "heart valve disease*" OR "rheumatic heart disease") |  |
| #3 | TS=("epidemiological study" OR "cohort study" OR cohort* OR "cross sectional study" OR "case control" OR incidence OR prevalence OR longitudinal OR prospective OR retrospective OR registry*) |  |
| #4 | DT=(Article) |  |
| #5 | #1 AND #2 |  |
| #6 | #5 AND #3 |  |
| #7 | #6 AND #4 | 54 |

| EMBASE | | |
| --- | --- | --- |
| #1 | 'alopecia areata'/exp OR 'alopecia areata':ti,ab OR 'alopecia circumscripta':ti,ab OR 'alopecia totalis':ti,ab OR 'alopecia universalis':ti,ab OR 'area celsi':ti,ab OR pelade*:ti,ab OR 'nonscarring hair loss':ti,ab OR 'scarring hair loss':ti,ab |  |
| #2 | 'cardiovascular disease'/exp OR 'heart disease'/exp OR 'myocardial infarction'/exp OR 'stroke'/exp OR 'hypertension'/exp OR 'arteriosclerosis'/exp OR 'atherosclerosis'/exp OR 'cardiotoxicity'/exp OR 'cardiomyopathy'/exp OR 'heart failure'/exp OR  cardiotoxic*:ti,ab OR 'cardiac toxic*':ti,ab OR 'cardiovascular disease*':ti,ab OR 'heart disease*':ti,ab OR 'ischemic heart disease*':ti,ab OR 'ischaemic heart disease*':ti,ab OR 'coronary occlusive disease*':ti,ab OR 'myocardial infarction*':ti,ab OR  'myocardial ischemia*':ti,ab OR 'stroke*':ti,ab OR 'heart attack*':ti,ab OR 'heart failure':ti,ab OR 'hypertension*':ti,ab OR 'arteriosclerosis':ti,ab OR 'atherosclerosis':ti,ab OR embolism*:ti,ab OR aneurysm*:ti,ab OR cardiomyopath*:ti,ab OR  pericarditis:ti,ab OR myocarditis:ti,ab OR endocarditis:ti,ab |  |
| #3 | 'epidemiology'/exp OR 'epidemiologic study'/exp OR 'cohort analysis'/exp OR 'longitudinal study'/exp OR 'case control study'/exp OR 'cross-sectional study'/exp OR 'follow up'/exp OR  epidemiolog*:ti,ab OR cohort*:ti,ab OR longitudinal:ti,ab OR prospective:ti,ab OR retrospective:ti,ab OR 'case control':ti,ab OR 'cross sectional':ti,ab OR prevalence:ti,ab OR incidence:ti,ab OR registry:ti,ab OR registries:ti,ab |  |
| #4 | 1 AND 2 AND 3 |  |
| #5 | 'review'/it OR 'editorial'/it OR 'letter'/it OR 'conference abstract'/it OR 'note'/it OR 'systematic review'/it OR 'meta analysis'/it |  |
| #6 | 'animal'/de NOT 'human'/de |  |
| #7 | 4 NOT 5 NOT 6 | 932 |

| Cochrane Library | | |
| --- | --- | --- |
| #1 | ("alopecia areata" OR "alopecia circumscripta" OR "alopecia totalis" OR "alopecia universalis" OR "area celsi" OR pelade OR "nonscarring hair loss" OR "scarring hair loss" OR alopecia) |  |
| #2 | (cardiotoxicity OR "cardiac toxicity" OR "cardiovascular disease" OR "heart disease" OR "ischemic heart disease" OR "ischaemic heart disease" OR "coronary occlusive disease" OR "myocardial ischemia" OR "myocardial infarction" OR "cardiovascular stroke" OR "heart attack" OR "heart failure" OR "cardiac failure" OR "myocardial failure" OR "angina pectoris" OR "cardiac valve disease" OR "heart valve disease" OR "valvular heart disease" OR "rheumatic heart disease" OR "essential hypertension" OR "idiopathic hypertension" OR "hypertensive disease" OR "hypertensive heart disease" OR "pulmonary embolism" OR "pulmonary heart disease" OR pericarditis OR endocarditis OR myocarditis OR cardiomyopathy OR arrhythmia OR "atrial fibrillation" OR stroke OR hemorrhage OR aneurysm OR embolism OR "arterial disease" OR "venous thrombosis" OR "cerebrovascular disorder" OR "brain infarction" OR "cerebral hemorrhage" OR hypotension OR "deep vein thrombosis" OR "varicose vein") |  |
| #3 | ("epidemiological study" OR "epidemiological studies" OR cohort OR "incidence study" OR "cross sectional study" OR "prevalence study" OR "case control" OR "follow up study" OR longitudinal OR prospective OR retrospective OR registry OR registries) |  |
| #4 | 1 AND 2 AND 3 | 211 |
